# Influence of thermodisinfection on microstructure of human femoral heads: duration of heat exposition and compressive strength

**DOI:** 10.1007/s10561-020-09832-5

**Published:** 2020-04-20

**Authors:** Christian Fölsch, Julian Dharma, Carlos Alfonso Fonseca Ulloa, Katrin Susanne Lips, Markus Rickert, Axel Pruss, Alexander Jahnke

**Affiliations:** 1 Department of Orthopaedic Surgery, Justus-Liebig-University Medical School, Klinikstrasse 33, 35392 Giessen, Germany; 2 Labarotory of Biomechanics, Department of Orthopaedic Surgery, Justus-Liebig-University Medical School, Klinikstrasse 29, 35392 Giessen, Germany; 3 Laboratory of Experimental Trauma Surgery, Justus-Liebig-University, Aulweg 128, 35392 Giessen, Germany; 4 University Tissue Bank, Institute of Transfusion Medicine, Charité University Medical School, Charitéplatz 1, 10117 Berlin, Germany

**Keywords:** Allogeneic bone, Human femoral head, Compression force, Microarchitecture bone, Thermodisinfection, Bone bank procedures

## Abstract

Allogeneic bone derived from living donors being necessary to match demand for bone transplantation and thermodisinfection of femoral heads is an established sterilization method. During the thermodisinfection the peripheral bone is exposed to maximum 86 °C for 94 min providing 82.5 °C within the center of the femoral head for at least 15 min. This study examined the compression force of the central and representative peripheral regions of native and thermodisinfected human femoral heads to observe wether different duration and intensity of heat exposure might alter mechanic behaviour. Slices from the equatorial region of human femoral heads were taken from each 14 native and thermodisinfected human femoral heads. The central area revealed a significantly higher compression force for native (*p* ≤ 0.001) and for thermodisinfected bone (*p* = 0.002 and *p* = 0.005) compared with peripheral regions since no relevant differences were found between the peripheral and intermediate areas themselves. A small reduction of compression force for thermodisinfected bone was shown since this did not appear significant due to the small number of specimens. The heat exposure did not alter the pre-existing anatomical changes of the microarchitecture of the native femoral heads from the center towards the peripheral regions. The heterogeneity of microstructure of the femoral head might be of interest concerning clinical applications of bone grafts since the difference between native and thermodisinfected bone appears moderate as shown previously. The different quantity of heat exposure did not reveal any significant influence on compression force which might enable thermodisinfection of preformed bone pieces for surgical indications.

## Introduction

Allogeneic bone is necessary to match the growing demand for bone replacement material in orthopaedic surgery. The transplantation of bone derived from femoral heads during hip replacement surgery might be regarded as gold standard (Ahmed et al. 2018). The morphology of the allogeneic cancellous bone is not exactly known before surgery and several bone bank procedures apply different physical and chemical methods for preparation since native bone preserves mechanic properties. Concerning the transmission of diseases thermodisinfection of femoral heads from living donors can be regarded as a save method (Pruss et al. 2003) since the impairment of mechanic properties up to 20% appears acceptable concerning its clinical use (Fölsch et al. 2012, 2015, 2016). The duration of heat application to the femoral head is determined by the necessary inactivation time for bacteria and viral agens for at least 15 min at 82.5 °C which limits the maximal diameter of the head of femur to 56 mm. The peripheral areas of the bone specimens are being exposed to a higher temperature for a longer time since the duration of the temperature within the centre depends on the size of the femoral head. Smaller specimens would increase their temperature early and different time of exposure to heat might influence mechanic parameters. A relevant effect of thermodisinfection on mineral structures cannot be assumed since heat application might influence collagen structures. Collagen crosslinks are important for bone fragility and mineral content might be more important for strength and stiffness since collagen changes with age could be reasonable to reduce bone toughness meaning bone failure (Burr 2002). Inter-site variations of bone architecture and the heterogeneity of the bone structure have to be considered (Bruyère Garnier et al. 1999; Djuric et al. 2013; Fölsch et al. 2015, 2016; Morgan et al. 2004).

Geometry, microarchitecture and bone mineral density of head and neck of femur as well as greater and minor trochanter region correlated significantly with compressive strength since structural parameters and bone mineral density were found equally related (Hansen et al. 2011). Specific alterations of bone structure in osteoarthritis have to be considered (Djuric et al. 2013; Chappard et al. 2006) since remodelling of bone structure is important for the integrity of the bone structure (Gentzsch et al. 2003). Harvesting cancellous bone from different areas of the femoral head might have an influence on the mechanic properties of the bone graft quality since density gradients within femoral heads had been described (Bruyère Garnier et al. 1999). The mechanic strength of the bone graft might be very important for some clinical applications and this could be relevant for harvesting and manufacturing of specially designed bone grafts. Therefore measurement of the microarchitecture of the femoral head using CT-scan might be of relevance for clinical application. Compression force applied to the vertical axis of bone cylinders is commonly used to test characteristics of bone fracture (Banse et al. 1996; Fölsch et al. 2016; Perilli et al. 2008; Wachter et al. 2001b) since the mode of failure showed the importance of shear stress (Nazarian et al. 2009).

Different mechanic properties related to individual anatomy and the location of the specimens in the head of femur independently from bone bank procedures should be of interest for clinical applications since thermodisinfection might additionally influence mechanic behaviour related to duration and intensity of heat exposure. The study was designed to examine mechanical strength at the center of the head of native and thermodisinfected human femora and at different centrifugal distances representative for all regions of the head of femur using compressive force. Manufacturing of cancellous bone transplants from the femoral head for surgical indications before performing the thermodisinfection could be an alternative during preparation in the operating theatre since the location of harvesting might be relevant for its clinical application. Histologic examination including collagen I staining was performed to examine the influence of thermodisinfection on osteoid, osteocytes and collagen structure.

## Methods

**Fig. 1:**
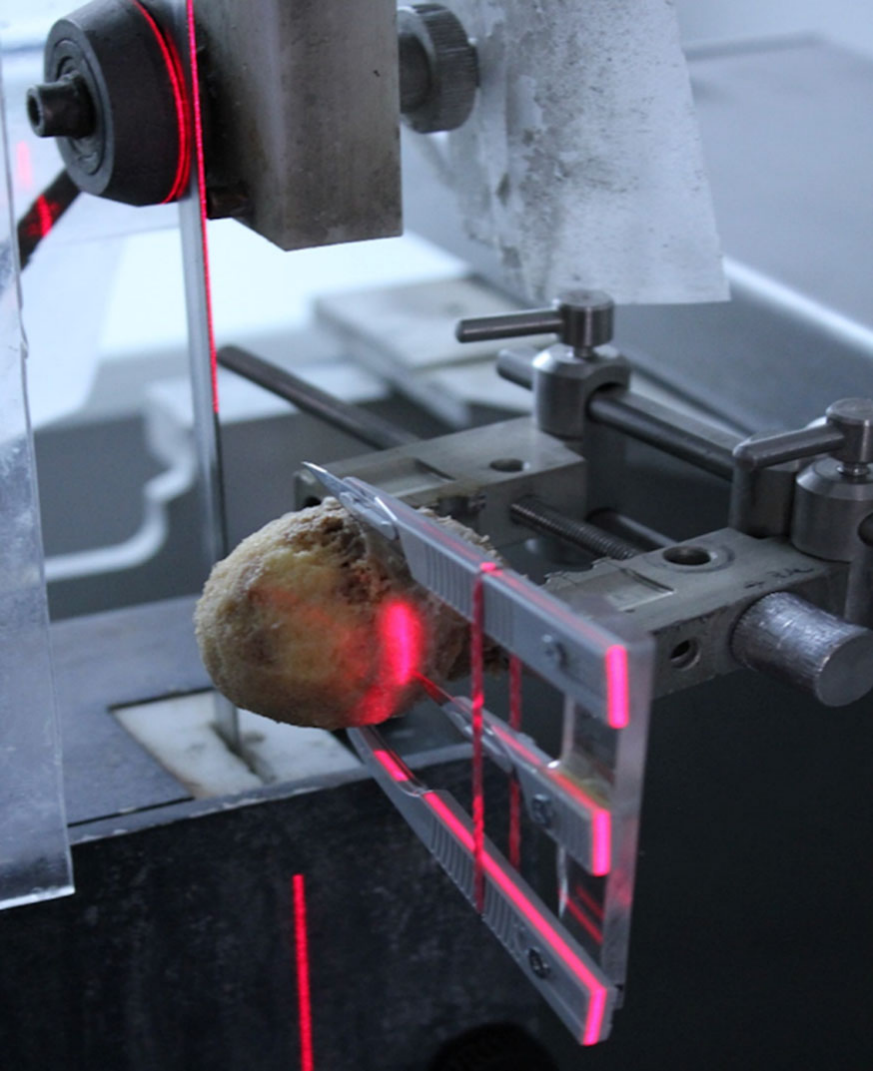
Positioning of the femoral head in the diamond saw using a self-leveling line laser and a positioning aid

**Fig. 2:**
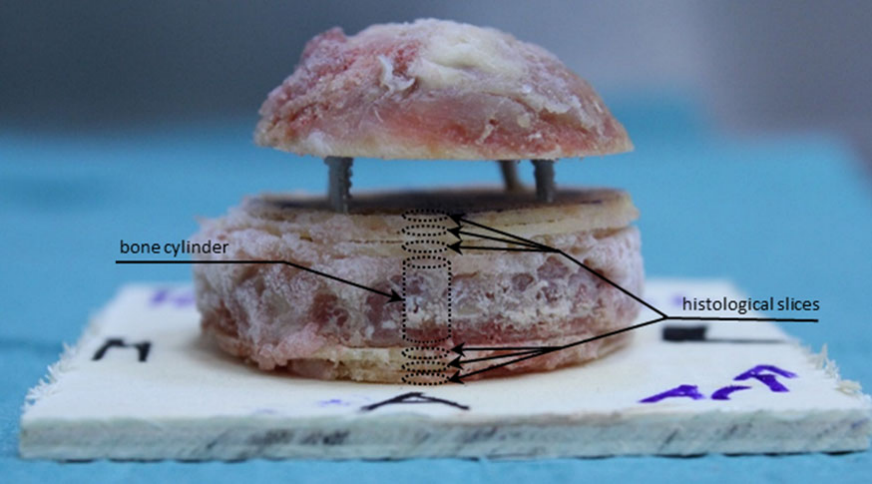
Reconstructed section slices being sorted and fixed on plywood board showing the bone cylinder for mechanical test and the adjacent smaller slices for histologic analysis

The necessary number of specimens within each group was calculated according to a previous study (Fölsch et al. 2016). After randomization of native and thermodisinfected specimens nine bone cylinders were taken from a slice of the equatorial region of each 14 native and 14 thermodisinfected human femoral heads provided by Telos Marburg using a diamond band saw (300CI, EXAKT, Norderstedt, Deutschland) (Fig. 1). The goal was to dissect a central disc with a height of 8 mm with the largest radius in the middle of the femoral head. From that slice 9 cylinders were obtained each for compression testing. Corresponding to the central disc slice three additional slices with a thickness of 1 mm each were harvested from the adjacent cranial and caudal area for histological examinations (Fig. 2). The anatomy of the femoral head including the central fovea allowed orientation revealing the longer medio-lateral diameter compared with the anterior–posterior direction. The anterior–posterior aspect was reconstructed using the triangular-shaped cross-sectional geometry of the remaining femoral neck. The orientation of the perpendicular direction of the saw towards the equatorial level of the head of femur was monitored by a self-leveling line laser (BALS01B, Timbertech_JAGO AG, Stuttgart, Germany) and a special designed device designated the largest diameter and the orientation of the femoral head (Fig. 1). The femoral heads were marked with a sterile surgical pen to allow reconstruction of the bone discs and the orientation of the femoral heads after the cuts had been made. Subsequently all force-controlled cuts were performed with the aforementioned diamond band saw under constant water cooling and with the aid of a drop weight with a mass of m = 350 g.

**Fig. 3:**
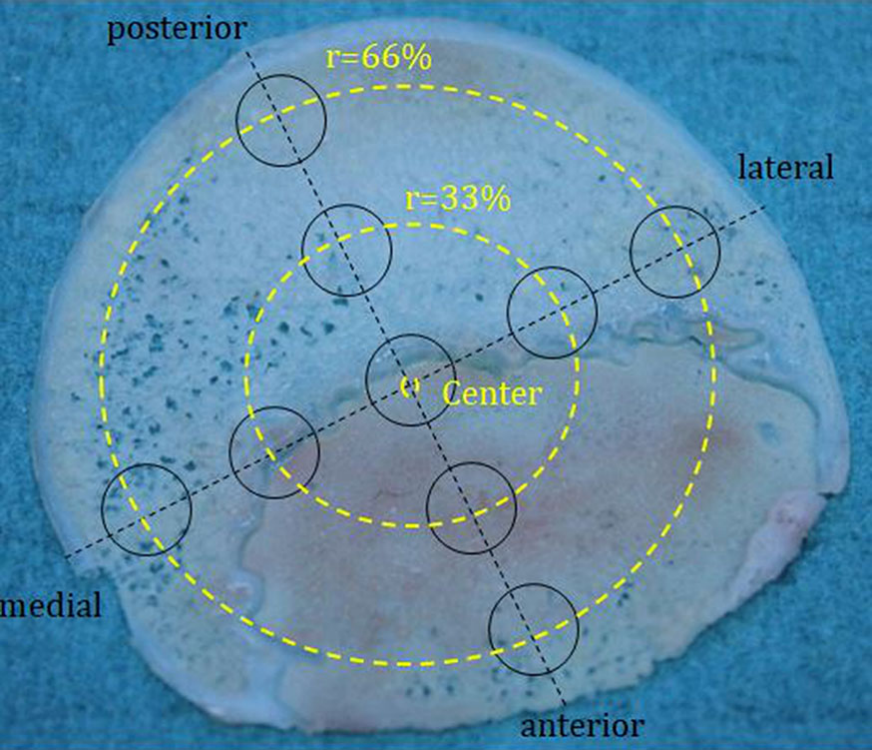
Exemplary orientation of the intermediate and peripheral bone cylinders in percent dependent on the femoral radius and the central cylinder

All bone slices were reassembled according to the prior marking lines and were fixed on a wooden board with the help of nails (Fig. 2). In accordance to the anterior–posterior and the medio-lateral diameter of the femoral head the intermediate cylinders were selected according to the 33% radius of the femoral head and the peripheral cylinders were taken as a function of the 66% radius of the femoral head measured from the center respectively (Fig. 3). Using a vernier caliper and taking into account the orientation of the femoral head, its center and according to the radius, the intermediate and peripheral center points of the bone cylinders were determined and marked with a sterile surgical pen. Subsequently the fixed bone discs were prepared with the hollow drill and a drilling machine (Metabo-BE1100, Nürtingen, Germany) which then resulted in a total of six bone slices with a height of 1 mm and one bone cylinder with a height of 8 mm with a diameter of 5 mm each (Figs. 2, 3).

All specimens were marked and cryopreserved at − 20 °C. Before further testing the bone specimens were warmed up in 21 ± 1 °C normal saline solution for 3 h (NaCl 0.9%, Braun Melsungen, Germany) within an incubator (2247383, Memmert GmbH&Co KG. Schwabach, Germany). Compression test measurements were then performed with a universal testing machine (Inspekt Table Blue 20kN, Hegewald&Peschke, Nossen, Germany) using an additional sensor with a registration range of 2 kN and a resolution of ± 1 N (Type 8402, Burster, Gernsbach, Germany) and a special fixation device reducing shear forces. The bone specimens were mounted into the testing machine leaving 2 mm distance to the sensor. The bone cylinders were exposed to axial compression until failure with a constant velocity of the testing machine of 2.5 mm/min. Test criterion was determined as the maximum compression limit load of the samples.

Bone specimens for histological examinations were embedded (Technovit 9100, Kulzer GmbH, 63450 Hanau, Germany) after fixation and dehydration. Following 5 µm slices were taken from each three slices with Rotationsmikrotom Leica RM2155 (Leica Biosystems Nussloch GmbH, 69226 Nussloch, Germany). Staining was done with toluidin-blue where cells and soft tissue would show different kinds of blue since cartilage matrix would be violet. Movat-pentachrom staining visualizes remodeling of bone showing chondral ossification since osteoid appears dark red. For visualization of collagen I immunohistochemic staining was performed with human polyclonal antibodies (Biologo, CO20111) and using Vectastatin Elite ABC-Kit, Nova-Red and Hämatoxilin 1 and 3. The specimens were fixed with DePex. The histologic examination was done descriptive for native and thermodisinfected bone and no further differentiation related to the location of the specimens was made.

### Statistical analysis

Data are presented as total values and averages (MEAN) with standard deviation (SD). The distribution for all specimens and between the two groups was examined with the Kolmogorow–Smirnow-Test. In order to investigate possible differences in the maximum compression characteristics of the bone cylinders as a function of the treatment (thermodisinfected vs. native) and taking into account the localization, the non-parametric Kruskal–Wallis test was used. The 95% confidence interval was calculated for the distribution of the compression force measurements. For pairwise comparison the Dunn-Bonferroni post hoc-test was applied. The significance level was adjusted due to multiple testing according to the Bonferroni correction. The level of significance was defined with α = 0.05.

## Results

**Fig. 4:**
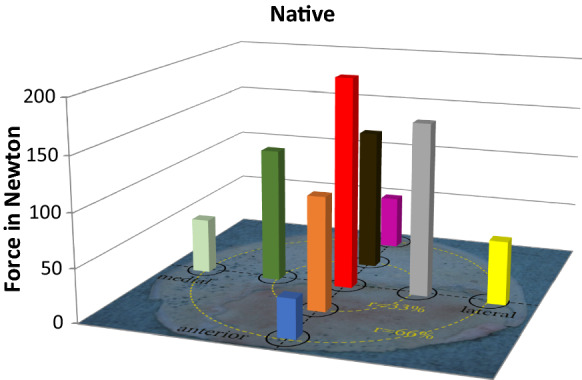
Influence of the localization of the native bone cylinders on the mechanical strength of the specimens

**Fig. 5:**
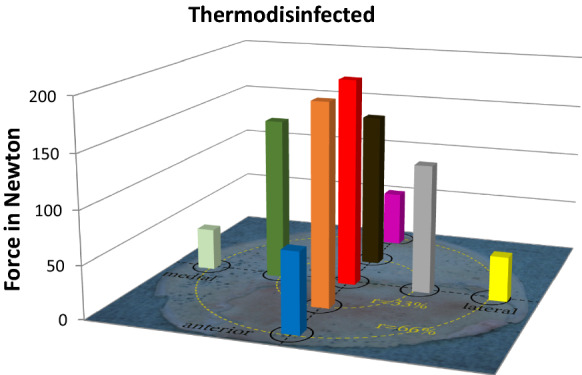
Influence of the localization of the thermodesinfected bone cylinders on the mechanical strength of the specimens

According to quality of bone specimens 124 cylinders from native and 123 cylinders from thermodisinfected femoral heads were included into the examination. One thermodisinfected cylinder had to be excluded as it already showed visual damage. From all measured cylinders the central specimens showed the highest compression force for native (214.7 N ± 136.1 N) (Fig. 4) and thermodisinfected (196.9 N ± 147.7 N) bone (Fig. 5) since the maximum compression force was reduced towards the peripheral areas of the slices taken from the femoral heads. The lowest compression force of the peripheral region decreased to 36.2 N ± 30.7 N in the native and to 42.1 N ± 30.5 N in the thermodisinfected group (Table 1). A significant reduction of compression force was shown between the central area and all peripheral regions for native bone since the significance level varied slightly between those regions (anterior and medial *p* < 0.001, posterior and lateral *p* = 0.001). For the thermodisinfected bone two out of four of the peripheral regions were significantly different from the central area of the femur (lateral *p* = 0.005 and medial *p* = 0.002) since the compression force in the peripheral anterior and posterior region showed relevant lower absolute values compared with the center and the values were higher compared with the corresponding native specimens (Table 1). A reduction of the compression force corresponding to more peripheral regions of the femoral head could be observed in native and thermodisinfected bone in similar pattern (Table 1). There was no significant difference between the four intermediate regions as well as none between the four peripheral areas of the native and thermodisinfected bone except the anterior and posterior intermediate region of thermodisinfected bone (*p* = 0.015). No significant differences were found between the central area and the intermediate area neither for native nor for thermodisinfected specimens since the high standard deviations have to be considered. Several significant differences of compression force were found between the intermediate and peripheral regions of native and thermodisinfected specimens (Tables 2, 3) since a relevant reduction of absolute values appeared (Table 1). The Kolmogorow-Smirnow-Test showed a comparable distribution for native and thermodisinfected bone. High standard deviations were found for compression forces for all measured bone cylinders and the 95% confidence interval for native bone from the center ranged from 170 N to 260 N and from 154 N to 240 N for thermodisinfected specimens.

**Table 1:** Compression force (N) including standard deviation at different areas of the femoral head in the equatorial region

Location bone specimens	Native bone	Thermodisinfected
Central area	$214.7\,{\text{N}} \pm 136.1\,{\text{N}}$	196.9 N ± 147.7 N
Anterior peripheral	$36.2\,{\text{N}} \pm 30.7\,{\text{N}}$	$74.8 \,{\text{N}} \pm 58.7\,{\text{N}}$
Anterior intermediate	$106.5\,{\text{N}} \pm 59.9\,{\text{N}}$	$189.5 \,{\text{N}} \pm 106.3\,{\text{N}}$
Posterior peripheral	$50.9\,{\text{N}} \pm 45.8\,{\text{N}}$	$53.1\,{\text{N}} \pm 35.0\,{\text{N}}$
Posterior intermediate	$134.0 \,{\text{N}} \pm 90.9\,{\text{N}}$	$147.5 \,{\text{N}} \pm 82.5\,{\text{N}}$
Lateral peripheral	$59.8 N \pm 53.3 N$	$42.1\,{\text{N}} \pm 30.5\,{\text{N}}$
Lateral intermediate	$163.0\,{\text{N}} \pm 103.7\,{\text{N}}$	$121.8 \,{\text{N}} \pm 90.8 \,{\text{N}}$
Medial peripheral	$52.1 \,{\text{N}} \pm 47.4\,{\text{N}}$	$39.6 \,{\text{N}} \pm 36.1\,{\text{N}}$
Medial intermediate	$126.4\,{\text{N}} \pm 47.9\,{\text{N}}$	$152.9\,{\text{N}} \pm 71.8\,{\text{N}}$

**Table 2:** Correlation between compression force with level of significance (*p* value) between intermediate and peripheral location of bone specimens from native bone

Intermediate region	Peripheral region	Significance
Medial	Anterior	0.003
Medial	Medial	0.043
Medial	Posterior	0.042
Lateral	Anterior	0.001
Lateral	Posterior	0.023
Lateral	Medial	0.024
Posterior	Anterior	0.012

**Table 3:** Correlation between compression force with level of significance (*p* value) between intermediate and peripheral location of bone specimens from thermodisinfected bone

Intermediate region	Peripheral region	Significance
Anterior	Lateral	0.001
Anterior	Medial	< 0.001
Posterior	Lateral	0.020
Posterior	Medial	0.008
Medial	Lateral	0.004
Medial	Medial	0.001
Medial	Posterior	0.040

**Fig. 6:**
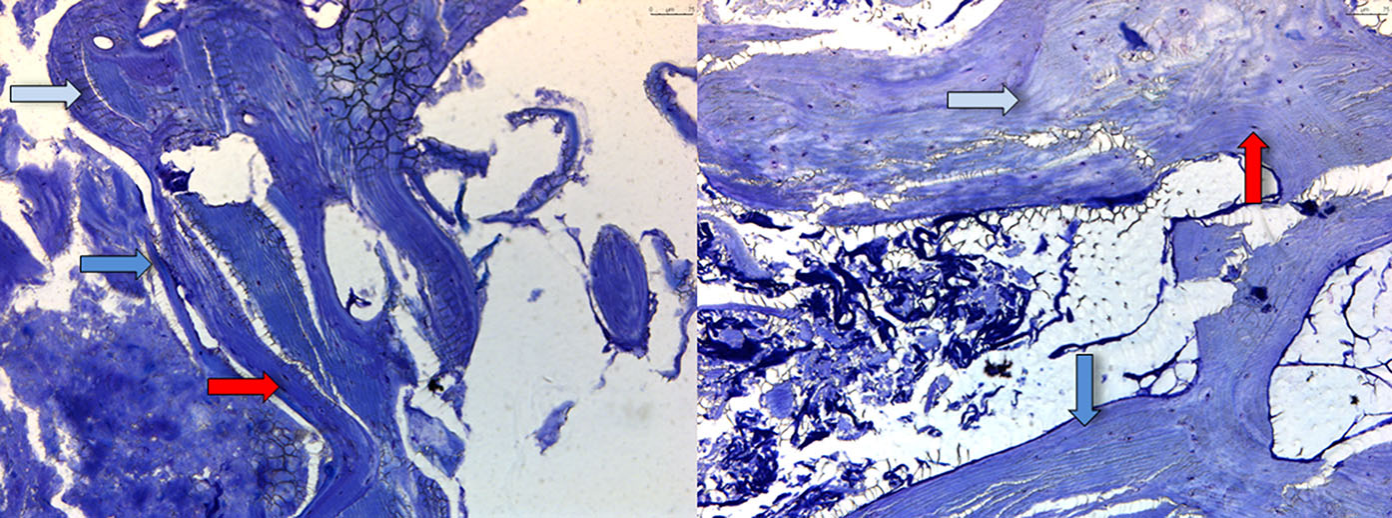
Native (left) and thermodisinfected (right) bone stained with toludine blue—O in ×10 magnification. Light blue arrows mark mineralized and dark blue arrows show not mineralized tissue. Red arrows point out osteocytes. (Color figure online)

**Fig. 7:**
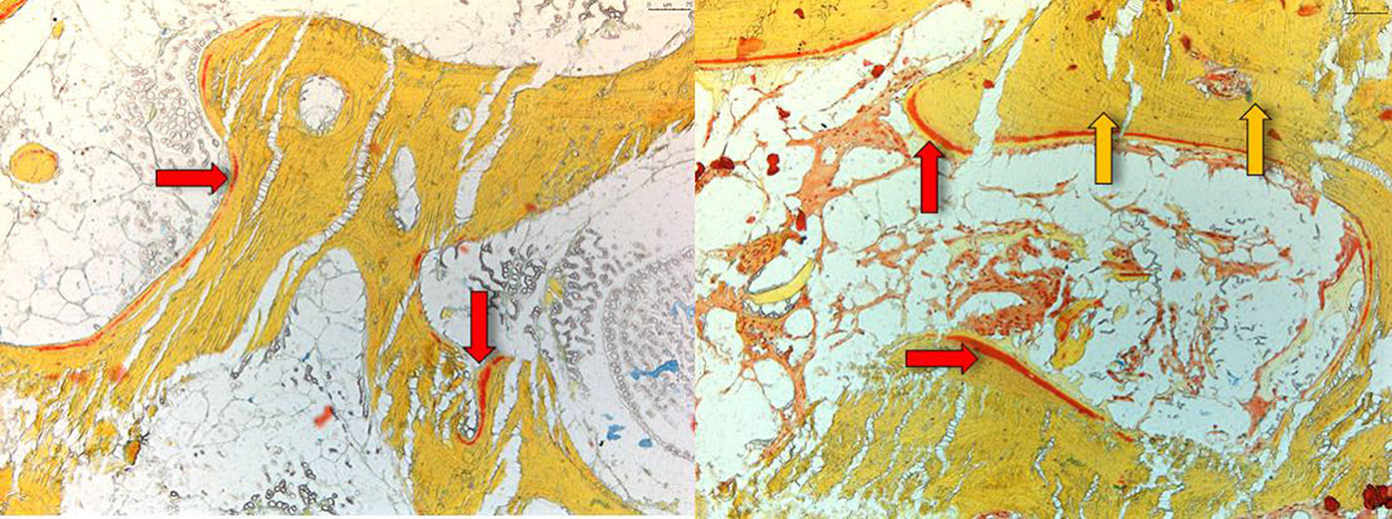
Native (left) and thermodisinfected (right) bone stained with movat-pentachrome in ×10 magnification. Red arrows show non mineralized and yellow arrows mark mineralized tissue. (Color figure online)

**Fig. 8:**
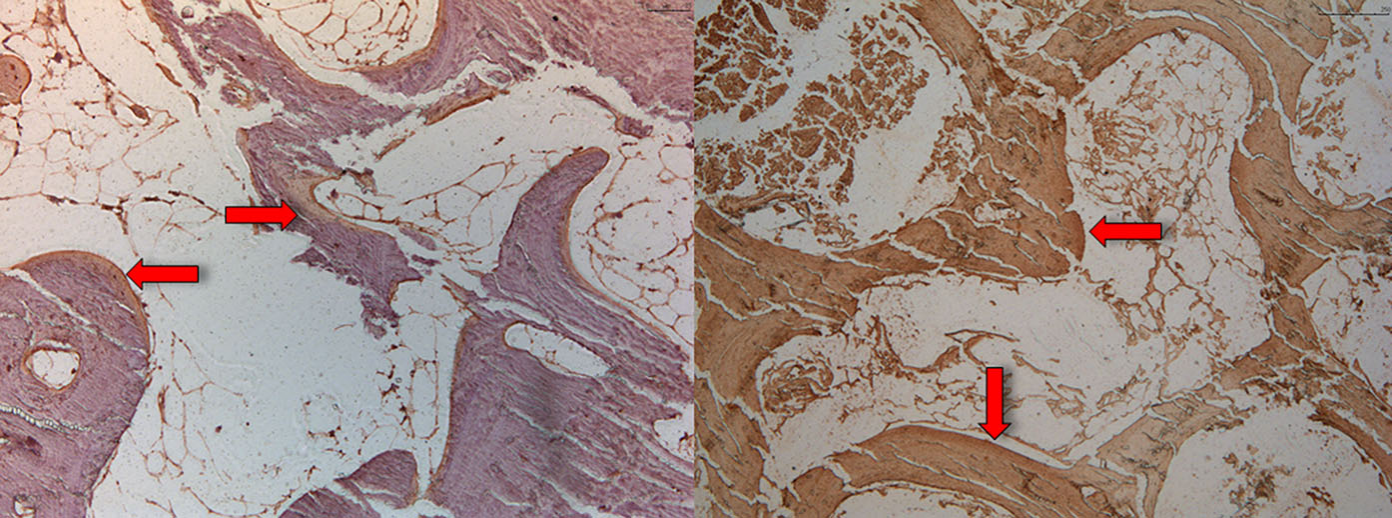
Native (left) and thermodisinfected (right) bone immunohistochemically stained by means of collagen I specific/hylase in ×10 magnification

The toluidin-blue staining revealed osteocytes in native and thermodisinfected bone specimens (Fig. 6). Few non-mineralized areas were found since a quantitative measurement was not feasible. The sections showed the mineralized hard tissue matrix in the blue shade brighter than the darker which were not mineralized. As expected the mineralized hard tissue matrix occupied a large part of the area with isolated non-mineralized areas. Osteocytes could be found on the cellular level in native and thermodisinfected bone since an exact comparison in terms of quantity was not feasible (Fig. 6). In movat-pentachrom staining non-mineralized areas of bone appeared red and the thermodisinfected bone showed more of those areas (Fig. 7). Collagen type I appears yellow which could not significantly be shown in both groups. Dark yellow areas predominate in both samples representing mineralized bone. Collagen fibers type I impressed bright yellow but were not clearly identifiable in both groups. The non-mineralized osteoid turned red during dyeing which signifies bone remodeling processes and the thermodisinfected preparation apparently showed more red areas since quantitative statements were not reliable due to the evaluation method but the staining indicated still active conversion processes after thermal treatment (Fig. 7). Immunohistochemic detection of collagen I was positive in native and thermodisinfected bone specimens since this appeared more intense for native bone and few osteocytes were found in the thermodisinfected bone and some more were shown in the native group (Fig. 8).

## Discussion

Bone quality of allogeneic femoral heads is relevant for transplantation but it is not routinely measured before transplantation. Intraindividual asymmetry of the proximal femur has been described (Laumonerie et al. 2018) but also no side-to-side difference was shown (Banse et al. 1996) since even geographic variability should be considered (Hsu et al. 2019). The femoral head diameter showed strong and more influence on mechanical properties than body weight (Sun et al. 2008) and femoral anteversion revealed an influence on the diameter of the neck of femur since femoral anteversion correlated with femoral head center difference (Dimitriou et al. 2016). Aging was found to be relevant for geometry of the proximal femur since the neck shifted in relative varus position with increasing age (Boymans et al. 2017). Independent of age the proximal femur exhibits an optimum structure due to mechanical loading according to Wolff´s law although anisotropic regions remain (Boyle and Kim 2011; Jang and Kim 2008). The adaptation of bone during aging and osteoarthritis induced by a decrease in tissue stiffness resulted in an almost constant stiffness in the main load bearing direction since the transversal stiffness decreased and an increase of tissue stiffness resulted in a higher stiffness in the main direction (van der Linden et al. 2004). This might be supported by the measurements of the compression force for native as well as thermodisinfected specimens which appeared significantly higher in the central compared with peripheral areas of the head of femur (Table 1, Figs. 4, 5). The measured values showed a wide range according to an expected anisotropy of the cancellous bone in multiple locations. The measured values and the standard deviations varied considerably between the native and thermodisinfected bone specimens from different locations since values appeared higher in thermodisinfected specimens in some regions of the femoral head since lower values would have been expected (Fölsch et al. 2015, 2016)(Table 1). Overall significant differences were found between the central region in native bone compared with all four peripheral locations and in thermodisinfected bone the measured values were slightly lower compared with the native bone since the pattern of reduction towards peripheral regions appeared comparable between native and thermodisinfected specimens (Table 1, Figs. 4, 5). Significant differences between the central region and the peripheral area of the femoral head were found in two out of four measured regions in the thermodisinfected bone (Table 1). The values measured were more than fourfold higher in the center. Significant reduction of compression force was found between several intermediate and peripheral areas of native (Table 2) and thermodisinfected bone (Table 3). No significant differences of compression force were measured between the intermediate and peripheral areas of native and thermodisinfected bone themselves except two regions of thermodisinfected bone. The small sample size together with the bone anisotropy apparently accounted for the high standard deviations. Previous studies had shown similar influence of thermodisinfection on different mechanical parameter of cancellous bone (Fölsch et al. 2015; Fölsch et al. 2016).

Cancellous bone was found to be more important to predict variability of DXA measurements of neck of femur and the trochanter region since the influence of the cortical bone appeared minor (Lundeen et al. 2001). Trabecular bone mineral density was shown to be detected reproducible by quantitative computertomography and correlated with biomechanical strength since the combination with geometry showed a higher correlation (Lang et al. 1997; Wachter et al. 2001a). Different tests might be performed to obtain relevant data of the mechanic properties of cancellous bone (Fölsch et al. 2016; Klinge 2013). We measured the compression force of multiple areas of cancellous bone from femoral heads. Architectural anisotropy of cancellous bone from femoral heads was found increased in fracture patients (Homminga et al. 2002) and those patients had a significantly decreased transverse stiffness resulting in increased mechanical anisotropy though the bone appeared weaker in non primary loading directions. This was also found in this group of osteoarthritis patients showing significant higher compression load in the central region of the femoral heads. The heterogeneity of cancellous bone was likely to be found in the regions of bone failure located in areas with minimum fraction of bone volume to trabecular volume located in the areas of compression failure (Perilli et al. 2008) since yield strength and stiffness of trabecular bone from femoral heads were best explained with the ratio of bone volume to trabecular volume and the trabecular anisotropy (Musy et al. 2017). Those areas of heterogeneity paired with reduced compression force were found in the peripheral regions of the femoral heads studied.

Cortical and trabecular geometry and volumetric bone mineral density, microarchitecture and areal bone mineral density correlated with maximum compressive strength (Hansen et al. 2011). The knowledge of relationship between bone microarchitecture and strength describes bone quality and might predict fracture risk and show bone remodeling process (van der Linden and Weinans 2007). Geometry of cortical and cancellous bone both contributed nearly similar to failure rate of cervical femoral fractures since densitometric variables measured with quantitative QCT were more predictive for failure than geometry (Bousson et al. 2006). Therefore the threedimensional structure of cancellous bone grafts which correlated with compression force from femoral heads should be considered and the correlating compression load was examined. The microarchitecture of the femoral head provided accurate discrimination between femoral heads from fractured and non-fractured femoral heads and the combination with bone mineral density improved the estimation of femoral neck fracture risk (Ollivier et al. 2013). The measurements of compression loads showed a high standard deviation in the peripheral regions corresponding to a considerable variability of cancellous bone in this study. Quantitative computed tomography allows differentiation between cortical and trabecular bone measuring true volumetric bone mineral density since increased areal bone mineral density was associated with decreased trabecular volume and increased cortical volume has to be considered (Amstrup et al. 2016). Elastic modulus of neck of femur was found lower for trabecular than for cortical tissue (Bayraktar et al. 2004) and microarchitecture of femoral head measured with micro-computed tomography was shown to be different for several parameters between fractured and not fractured specimens (Greenwood et al. 2018) since the correlation between bone mineral density measured with regional quantitative CT and failure was higher in the femoral head compared with the neck of femur (Huber et al. 2008). Therefore computertomographic examination of microstructure of allogeneic bone transplants might be considered before transplantation to characterize its bone structure. Micro-CT scan was shown to provide a three dimensional view out of a two dimensional histology (Katsamenis et al. 2019).

In Osteoarthritis the neck of femur showed reduced anisotropy especially in the inferomedial neck and higher trabecular connectivity since all micro-architectural parameters displayed significant regional heterogeneity (Djuric et al. 2013). These findings seem to correlate with the high standard deviations of the compression load measured in different areas of the femoral head in this study. Subchondral bone changes were mainly observed in advanced osteoarthritis though these changes seemed to be secondary to cartilage deterioration (Chappard et al. 2006). This might be an additional reason for reduced compression load in the peripheral regions.

Changes of microarchitecture according to osteoporosis have to be considered which might appear within head of femurs from living donors since it seems to be unknown wether patients suffer from osteoporosis and osteoarthritis simultaneously (Sun et al. 2008). Correlation between fracture toughness and microarchitecture parameters of osteoporotic cancellous tissue were shown for fractured neck of femur and should improve bone mineral density measurement alone (Greenwood et al. 2015). The Singh index might be useful to describe trabecular structures of the femoral head and neck (Singh et al. 1970) since no significant association of that index with trabecular volume was found (Patel and Murphy 2006). Increase of bone remodeling and changes of morphology of subchondral bone were found in femoral heads with osteoporosis (Bobinac et al. 2013) and subchondral bone from overweight patients with osteoarthritis showed reduced trabecular thickness and an increase in type I collagen compared to normal weight patients with osteoarthritis (Philp et al. 2017). The inferomedial neck of femur showed increased anisotropy and variability of bone volume fraction and in case of neck of femur fracture superolateral almost all parameter were different as the fracture showed a lower trabecular volume fraction indicating the anisotropy of the changes of the architecture in osteoporosis and thinning of trabeculae (Milovanovic et al. 2012) which needs to be considered in judging the results of the mechanic tests in the present study. Patients with spontaneous femoral fractures exhibited lower bone density than the asymptomatic controls (Narayanan et al. 2019). Geometrical measurement combined with bone mineral density of head of femur and neck of femur improved prediction of bone failure (Yang et al. 2014a) and bone mineral content as well as bone mineral density and structural parameters correlated significantly with failure load of proximal femur (Bauer et al. 2006).Volumetric bone mineral density and apparent cortical thickness discriminated hip fracture on the opposite femur independently of areal bone mineral density by DXA (Yang et al. 2014b). The relevance of the mentioned parameter of bone structure seems important for the behaviour of bone grafts harvested from human heads of femur.

The results of our examinations showed a reduction of compressive force due to thermodisinfection which was comparable with previous examinations (Fölsch et al. 2015, 2016). This does not seem to be of clinical relevance if spongy bone bone grafts are used as non weight bearing filling material. According to the number of specimens in the present study there was no significant difference of compression load between native and thermodisinfected cancellous bone since we had described diminished mechanical resistance for shears stress and bending until failure previously (Fölsch et al. 2016). Thermodisinfection seems to alter compression force and other mechanic properties comparably (Fölsch et al. 2015, 2016) (Table 1). Osteocytes were shown in native and thermodisinfected bone (Fig. 6) since apparently reduced collagen I staining was found in thermodisinfected cancellous bone compared with native bone (Fig. 7). The interpretation has to consider a broad variety of interindividual and intraindividual cancellous bone morphology resulting in relevant standard deviations throughout different examinations (Fölsch et al. 2015, 2016). The significantly better mechanic quality measured by compression force within the center respectively the axis of the head of femur compared with the peripheral regions might be of relevance for harvesting bone from the head of femur and also for fixation of implants. The correlation with the neck of femur morphology described by Singh might be considered (Singh et al. 1970; Wachter et al. 2001a). The thermodisinfection procedure did no have an influence on the distribution of the mechanic properties with reduced compression stability from the center towards the peripheral regions of the femoral heads since a small reduction of compression force was found in a linear manner (Table 1, Figs. 4, 5). An underlying anatomic reason should be relevant for the reduction of compression force directed towards the periphery. Significant differences between the central area and the peripheral located areas of the femoral heads were shown and reduced absolute values were also found in the adjacent intermediate areas since heterogeneity of specimens resulted in high standard deviations (Table 1). The reduction of the compressive force of the cancellous bone might be expected to diminish gradually from the central region to the surface of the femoral heads also showing significant differences between the intermediate and peripheral regions (Tables 2, 3) according to underlying anatomical changes in native and thermodisinfected specimens.

The intensity of exposure to thermodisinfection with a higher and longer lasting temperature in the peripheral areas of the femoral heads did not show an additional negative effect on compressive force as the mechanic behaviour showed a similar pattern of reduction in the native bone. The maximum value of compression force appeared reduced following thermodisinfection and the measured absolute values of compression force showed a wide range according to the anisotropy of the bone similar to previous studies (Fölsch et al. 2015, 2016) since high standard deviations and number of specimens might influence the level of significance. Further examination of the alteration of collagen I following thermodisinfection might be useful. The morphology of bone grafts including changes of collagen seems to be relevant for osteoconduction and transport of cytokines and therefore is of great importance for healing and creeping substitution processes. The anatomical effect of reduction of compressive force from the center towards the peripheral areas of the femoral heads appeared independently from native or thermodisinfected specimens (Tables 1, 2, 3). Regarding the absolute values a relevant reduction was shown for all peripheral regions of the thermodisinfected and native bone since a significant difference was found in all native and in two out of four thermodisinfected specimens since significant change was found between several intermediate and peripheral regions (Tables 2, 3). The significant different mechanical properties of the femoral heads might be of relevance for harvesting bone for transplantation. The selection of bone graft from different areas of the femoral head might affect bone quality considerably more than the mechanical influence of thermodisinfection. Anatomical changes of microarchitecture of cancellous bone might be relevant regarding the surgical procedure. Exposure of cancellous bone is limited to a maximum temperature of 86 °C in the peripheral areas during 94 min which does not further impair the mechanic poperty of human femoral head cancellous bone compared with heat exposure to 82.5 °C in the center for 15 min. No different mechanical alteration related to the size of the thermodisinfected femoral head should be expected. Thermodisinfection of bone transplants manufactured for surgical procedures might be suitable and this could avoid processing of bone during surgery.

## Conclusion

Thermodisinfection did not significantly reduce compression force of cancellous bone from human femoral heads but showed an apparent reduction of absolute values similar to previous studies since collagen I staining appeared to be diminished for thermodisinfected bone. A comparable reduction of absolute compression load values was found consecutively from the center towards the peripheral areas for native and thermodisinfected bone indicating underlying common anatomical changes of microarchitecture. The central region of the femoral head revealed significantly higher compression force compared with peripheral areas of the equatorial region for native and thermodisinfected bone since no additional adverse effects of higher and longer heat exposition at the peripheral areas of the femoral heads were observed. Therefore thermodisinfection of manufactured bone transplants for surgical procedures might be useful and related to expected heterogeneity of the bone computed tomographic assessment might give a useful impression of the microstructure of the bone transplant to be chosen.
